# Age-Related Differences in Amygdala Activation Associated With Face Trustworthiness but No Evidence of Oxytocin Modulation

**DOI:** 10.3389/fpsyg.2022.838642

**Published:** 2022-06-23

**Authors:** Tian Lin, Didem Pehlivanoglu, Maryam Ziaei, Peiwei Liu, Adam J. Woods, David Feifel, Håkan Fischer, Natalie C. Ebner

**Affiliations:** ^1^Department of Psychology, University of Florida, Gainesville, FL, United States; ^2^Kavli Institute for Systems Neuroscience, Norwegian University of Science and Technology, Trondheim, Norway; ^3^Queensland Brain Institute, The University of Queensland, Brisbane, QLD, Australia; ^4^Department of Clinical and Health Psychology, Center for Cognitive Aging and Memory, University of Florida, Gainesville, FL, United States; ^5^Department of Psychiatry, University of California, San Diego, San Diego, CA, United States; ^6^Department of Psychology, Stockholm University, Stockholm, Sweden; ^7^Stockholm University Brain Imaging Centre (SUBIC), Stockholm, Sweden; ^8^Florida Institute for Cybersecurity Research, University of Florida, Gainesville, FL, United States; ^9^Department of Aging and Geriatric Research, Institute on Aging, University of Florida, Gainesville, FL, United States; ^10^The Evelyn F. and William L. McKnight Brain Institute (MBI), University of Florida, Gainesville, FL, United States

**Keywords:** face trustworthiness, aging, amygdala, fMRI, oxytocin

## Abstract

The amygdala has been shown to be responsive to face trustworthiness. While older adults typically give higher face trustworthiness ratings than young adults, a direct link between amygdala response and age-related differences in face trustworthiness evaluation has not yet been confirmed. Additionally, there is a possible modulatory role of the neuropeptide oxytocin in face trustworthiness evaluation, but the results are mixed and effects unexplored in aging. To address these research gaps, young, and older adults were randomly assigned to oxytocin or placebo self-administration *via* a nasal spray before rating faces on trustworthiness while undergoing functional magnetic resonance imaging. There was no overall age-group difference in face trustworthiness ratings, but older compared to young participants gave higher trustworthiness ratings to ambivalently untrustworthy-looking faces. In both age groups, lower face trustworthiness ratings were associated with higher left amygdala activity. A comparable negative linear association was observed in right amygdala but only among young participants. Also, in the right amygdala, lower and higher, compared to moderate, face trustworthiness ratings were associated with greater right amygdala activity (i.e., positive quadratic (U-shaped) association) for both age groups. Neither the behavioral nor the brain effects were modulated by a single dose of intranasal oxytocin administration, however. These results suggest dampened response to faces with lower trustworthiness among older compared to young adults, supporting the notion of reduced sensitivity to cues of untrustworthiness in aging. The findings also extend evidence of an age-related positivity effect to the evaluation of face trustworthiness.

## Introduction

Interpersonal trust is an important feature in human society and serves as a foundation for social relationships as well as economic and political activities (Van Lange, 2015). The trustworthiness that is perceived based on an individuals’ face (i.e., face trustworthiness), while not necessarily informative about the individuals’ actual status of trustworthiness, has been shown to affect interaction with the individual (Van’t Wout and Sanfey, 2008). Face trustworthiness evaluation can take place automatically and implicitly (Winston et al., 2002; Engell et al., 2007), with as minimal an exposure as 100 ms (Willis and Todorov, 2006).

Empirical evidence supports that the amygdala is involved in face trustworthiness evaluation (Bzdok et al., 2011; see Santos et al., 2016 for a meta-analysis). For example, individuals with bilateral amygdala lesions showed impaired evaluation of face trustworthiness (Adolphs et al., 1998). In healthy adults, lower face trustworthiness was associated with greater amygdala response (Winston et al., 2002; Engell et al., 2007). In addition to this negative linear trend, a positive quadratic (U-shaped) association was observed in that high and low (compared to moderate) face trustworthiness was correlated with greater amygdala response (Said et al., 2009; Mattavelli et al., 2012; Freeman et al., 2014), suggesting that amygdala may also generally respond to information with higher emotional salience (Sander et al., 2003; Ziaei et al., 2017).

These previous neuroimaging studies, however, have comprised young adults only, but there is emerging evidence of age-related differences in face trustworthiness evaluation, in that older adults may rate faces overall as more trustworthiness than young adults (Zebrowitz et al., 2013, 2017). This age effect was particularly pronounced for untrustworthy-looking faces, while young and older adults gave comparable ratings for trustworthy-looking faces (Castle et al., 2012; Zebrowitz et al., 2017; Cassidy et al., 2019; see Bailey and Leon, 2019 for a meta-analysis). This observation is consistent with findings that advanced age was associated with decreased sensitivity to deceptive cues (Ruffman et al., 2012; Frazier et al., 2021; see also Ebner et al., 2022, for a summary) and evidence of an age-related positivity effect (i.e., observed in the form of reduced negativity/increased positivity in older vs. young adults’ attention and memory; Carstensen et al., 2011; Pehlivanoglu and Verhaeghen, 2019; Ziaei et al., 2019, 2021). Based on these considerations, in the present study, we hypothesized that older compared to young participants would give higher trustworthiness ratings to faces overall, with this age-related difference particularly pronounced for less trustworthy-looking faces (*Hypothesis 1*).

Also, previous studies suggest age-related differences in amygdala response associated with face trustworthiness evaluation. Zebrowitz et al. (2018) explored age-related differences in amygdala response and found a trend wise negative linear association between amygdala activity and face trustworthiness in young adults, but older adults showed a trend wise positive quadratic relationship (i.e., high and low compared to moderate face trustworthiness was associated with greater amygdala activity). These trends were, however, not statistically significant when tested in each age group individually. Absence of significant age-related effects in Zebrowitz et al.’s study may have been related to the methodological approach used for defining regions of interest (ROIs). Specifically, Zebrowitz et al. (2018) defined amygdala as ROI based on an anatomical mask (Desikan et al., 2006). Their methodological approach therefore assumed that all voxels in the amygdala respond equally to face trustworthiness. Previous work, however, had demonstrated that only certain regions within the amygdala were responsive to face trustworthiness (Winston et al., 2002; Engell et al., 2007; Todorov, 2008; Said et al., 2009). Thus, analysis based on functional localization to identify regions within the amygdala that respond to face trustworthiness, rather than submitting all amygdala voxels to the analysis, may be more reliable and sensitive to determine age-related differences in the association between face trustworthiness and amygdala activity.

Applying this targeted approach in the present study, we hypothesized that young participants would show greater amygdala activity to untrustworthy- relative to trustworthy-looking faces while this effect would be reduced (or would not be present) in older participants (i.e., reduced (or no) *negative linear effect* of face trustworthiness on amygdala activity in older participants; *Hypothesis 2*). At the same time, based on evidence of relatively preserved ability to process emotionally salient information with advanced age (Leclerc and Kensinger, 2008), we predicted that both high and low (relative to moderately) trustworthy-looking faces would be associated with greater amygdala activity in both young and older participants (i.e., *positive quadratic (U-shaped) effect* of face trustworthiness on amygdala activity in both age groups; *Hypothesis 3*).

A separate line of work suggests a possible role of the nine amino acid peptide oxytocin on trust-related decision making and behavior, including face trustworthiness evaluation (Van IJzendoorn and Bakermans-Kranenburg, 2012; but see Keech et al., 2018). For example, a single dose of intranasal oxytocin, to trigger central modulation (Born et al., 2002), enhanced trust to others (Baumgartner et al., 2008; Mikolajczak et al., 2010; but see Declerck et al., 2020). Even more directly relevant to the present study, a single dose of intranasal oxytocin resulted in higher trustworthiness (and higher attractiveness) ratings to faces from strangers (Theodoridou et al., 2009, but see Lambert et al., 2014; Grainger et al., 2019).

Oxytocin-related enhancement of trust has been discussed as reflecting increased willingness to accept social risk (Kosfeld et al., 2005); and is in line with the prosocial hypothesis, which proposes that oxytocin increases attention to positive but not negative information, thus promoting affiliative prosocial behaviors (Meyer-Lindenberg, 2008; MacDonald and MacDonald, 2010). Intranasal oxytocin administration furthermore resulted in reduced amygdala activation to fearful faces (Kirsch et al., 2005); possibly reflecting anxiolytic properties of oxytocin (see also Labuschagne et al., 2010 for oxytocin’s anxiolytic properties in study on individuals with generalized social anxiety disorder). In line with both these accounts, oxytocin may reduce amygdala response associated with untrustworthy-looking faces.

Intranasal oxytocin has also been shown to promote altruistic behavior to in-group members but results in defensive aggression to members of the out-group (De Dreu et al., 2010). This social salience hypothesis proposes that oxytocin modulates the salience of social cues, regardless of their valence (Olff et al., 2013; Shamay-Tsoory and Abu-Akel, 2016). Further supporting this account is evidence of an oxytocin-related memory enhancement for faces (i.e., social stimuli) relative to objects (i.e., non-social stimuli; Rimmele et al., 2009). This oxytocin-related memory advantage for faces was accompanied by elevated attention to eyes (Guastella et al., 2008) and eye gaze direction (Tollenaar et al., 2013), suggesting that oxytocin facilitates attention to socially meaningful information.

Of note, this prior work has been done in young adults. Knowledge on age moderation of oxytocin effects, while limited, point to possible age-differential effects of intranasal oxytocin on social-cognitive processes (Campbell-Smith et al., 2015; Horta et al., 2019; Frazier et al., 2021; but see Grainger et al., 2018; also see Horta et al., 2020 for a recent overview). However, given the currently still small knowledge base, we refrained from formulating specific directional hypotheses pertaining to age-related differences in intranasal oxytocin modulation on face trustworthiness ratings and associated amygdala activity. In line with the social salience hypothesis that oxytocin draws attention to important social cues, one could propose that elevated oxytocin levels may increase older adults’ attention to cues of untrustworthiness in faces (and thus enhance their amygdala response to these faces), as negative facial signals that, while highly diagnostic, older adults may ignore otherwise given an age-related positivity bias.

## Materials and Methods

### Participants

This analysis was part of a larger project (see Ebner et al., 2015, 2016; Lin et al., 2018; Horta et al., 2019; Plasencia et al., 2019; Frazier et al., 2021). The current analysis sample comprised 98 of the originally recruited 105 participants,^1^ with 48 young (*M* = 22.43 years, *SD* = 2.97, 18–31 years, 48% females) and 50 older (*M* = 71.08 years, *SD* = 4.98, 63–81 years, 58% females) adults. Using G*Power, sensitivity analyses showed that with the current sample size and *p* = 0.05 as type I error threshold, we had 80% power to detect a small effect (Cohen’s *f* = 0.18; Cohen, 2013) for the interaction between age group and face trustworthiness level on trustworthiness ratings (*Hypothesis 1*); and 80% power to detect small effects (Cohen’s *f*^2^ = 0.13; Cohen, 2013) for the linear and quadratic trends of face trustworthiness and their interactions with age group on amygdala activity (*Hypothesis 2* and *3*). In addition, we had 80% power to detect a small effect (Cohen’s *f* = 0.22; Cohen, 2013) for the interaction between age group, treatment group, and face trustworthiness level on amygdala activity (i.e., exploratory analysis for oxytocin modulation).

All participants were English-speaking, White, with no history of neurological or psychiatric disorder, and with normal or corrected-to-normal vision. All older participants completed the Telephone Interview for Cognitive Status (TICS; Brandt et al., 1988; *M* = 35.47, *SD* = 2.42, Min = 30, Max = 42; cut off < 30) as screening for cognitive impairment. Twenty-six young (46% females) and 26 older (54% females) participants were randomly assigned to self-administer 24 international units (IUs) oxytocin *via* nasal spray and 22 young (50% females) and 24 older (63% females) participants to self-administer 24 IUs of a placebo nasal spray.

As shown in Table 1, overall, young participants performed better in the cognitive tasks (i.e., higher processing speed measured by the WAIS-R Digital Symbol Substitution Test (Wechsler, 1981) and better short-term memory measured by the Rey Auditory Verbal Learning Test (Rey, 1964) than older participants. The age groups also differed in positive affect with higher positive affect scores in older than young participants measured by the Positive Affect Negative Affect Scale (Watson et al., 1988; Röcke et al., 2009).

**TABLE 1:** Sample description: Mean (standard deviation) and age group as well as treatment group comparisons in demographic, health/biomarker, cognition, personality, and socioemotional measures.

	Young participants		Older participants		Age	Treatment	Interaction
Construct	Placebo	Oxytocin	Placebo	Oxytocin	η^2^*_*p*_*	η^2^*_*p*_*	η^2^*_*p*_*
	Mean (*SD*)	Mean (*SD*)	Mean (*SD*)	Mean (*SD*)			
**Demographics**							
Chronological age	22.81 (3.29)	22.01 (2.70)	70.73 (4.73)	71.42 (5.29)	**0.97**	<0.001	0.01
Education	15.86 (2.78)	15.19 (2.00)	16.20 (2.83)	16.96 (3.42)	0.04	<0.001	0.02
**Health/biomarker**							
Physical health	8.33 (1.28)	8.65 (1.06)	8.66 (1.07)	8.32 (1.03)	<0.001	<0.001	0.02
Mental health	8.57 (1.08)	8.46 (1.33)	9.08 (0.91)	8.63 (1.41)	0.02	0.01	0.01
Plasma oxytocin	795.85 (124.82)	806.46 (147.05)	777.30 (118.15)	790.13 (129.91)	0.01	0.002	<0.001
**Cognition**							
Processing speed	66.57 (12.38)	62.81 (7.95)	45.60 (8.70)	44.16 (9.00)	**0.53**	0.02	0.004
Short-term memory	9.38 (1.83)	9.00 (2.15)	7.68 (2.48)	7.32 (2.58)	**0.12**	0.01	<0.001
**Socioemotional functioning**							
Positive affect	2.83 (0.70)	2.83 (0.65)	3.57 (0.54)	3.34 (0.56)	**0.21**	0.01	0.01
Negative affect	1.26 (0.32)	1.15 (0.23)	1.18 (0.27)	1.28 (0.37)	0.002	<0.001	0.03

*Chronological Age and Education were reported in years. Physical Health “Please rate your general physical health” and Mental Health “Please rate your general mental health/mood” were measured by single self-report items on a scale from 1 (Poor) to 10 (Excellent). Plasma Oxytocin Levels (in picogram/milliliter) were determined using an Enzyme Immunoassay from Enzo Life Science, Inc. (Farmingdale, New York; see Plasencia et al., 2019, for details). Processing Speed was measured by the WAIS-R Digital Symbol Substitution Test (Wechsler, 1981). Short-term Memory was measured by the Rey Auditory Verbal Learning Test (Rey, 1964). Positive and Negative Affect were measured by the Positive Affect Negative Affect Scale (Watson et al., 1988; Röcke et al., 2009). SD, Standard Deviation. Bold indicates significant effect (p < 0.05).*

### Procedure

The present study used data from a larger project that comprised three test sessions: (1) a phone screen (about 30 min) to determine study eligibility (for drug administration as well as MRI) and to collect background demographic and health information; (2) an in-person screen visit (about 45 min), in which participants gave written informed consent, provided blood and urine samples, completed short questionnaires, and underwent a brief general health evaluation with the study clinician; and (3) an in-person full study visit lasting about 3 h, approximately 2–10 days after the in-person screen visit, during which participants self-administered the study drug and completed the fMRI task reported in this paper (as well as other (f)MRI and questionnaire measures reported elsewhere; Ebner et al., 2015, 2016; Horta et al., 2019; Frazier et al., 2021).

All study sessions were conducted by trained research staff and were completed at the Department of Psychology, the Institute on Aging, and the McKnight Brain Institute at the University of Florida. In-person visits started at about 9 a.m., given the fasting blood draw and diurnal hormone cycles. Participants were instructed to stay hydrated and to avoid caffeine and substance use 24 h before the in-person visits and abstain from food, exercise, and sexual activity at least 2 h before the visits. The study protocol was approved by the University of Florida Institutional Review Board (IRB#39-2013) and registered as a clinical trial with ClinicalTrials.gov (NCT01823146). Only measures considered in the present data analysis are described in detail below.

Short cognitive tasks as well as brief personality and socioemotional questionnaires were completed during the in-person screen visit and are presented in Table 1. Positive and negative affect was assessed at the start of the in-person full study visit (see Table 1 for details). This was followed by saliva sampling and self-administration of 24 IUs (one puff per nostril; IND #100,860) of either oxytocin or a placebo, which contained the same ingredients as the oxytocin spray except for the oxytocin. Treatment assignment was randomized and double-blinded, monitored by the dispensing pharmacy, and followed recommendations for standardized intranasal oxytocin administration (Guastella et al., 2013). Before self-administration, all pre-menopausal women completed a pregnancy test to assure safety.

Before entering the MRI scanner, participants underwent another MRI safety screening, received instructions on the scanning procedure, and practiced the tasks they were working on inside the scanner. The scanning session started about 45 min after drug self-administration, with brief anatomical scans and a 15-min decision-making paradigm (Frazier et al., 2021), before participants completed the *Face Trustworthiness Rating Task* analyzed for this study (see detailed description below). This was followed by a facial expression identification task (Horta et al., 2019), an 8-min resting state scan (Ebner et al., 2016), and a series of socioemotional questionnaires (Ebner et al., 2015), post-scan, debriefing, and compensation procedures outside the scanner.

### Face Trustworthiness Rating Task

**FIGURE 1:**
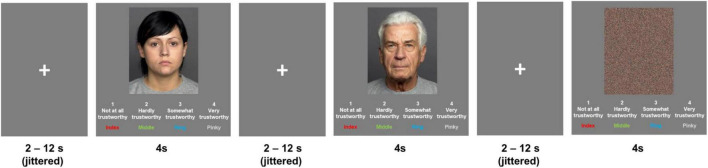
Face trustworthiness rating task: sample stimuli and trial timing. Facial image sources: FACES database (Ebner et al., 2010). Reproduced with permission.

As illustrated in Figure 1, each trial started with a jittered fixation cross (range: 2–12 s, mean = 4 s), followed by an image (either a face or a scrambled face) presented in the center of the screen for 4 s, with the rating scale and respective finger use for the response presented below the image. Participants were asked to rate the trustworthiness of each face on a four-point scale (1 = *Not at all trustworthy* to 4 = *Very trustworthy*) using an MRI compatible 4-button box, while the face was on the screen, or press any of the four buttons while the scramble was on the screen. Participants’ response and reaction time for each trial were recorded. The task was presented on Experiment Builder (SR Research), separated into two functional runs (about 7 min/run) to reduce participant burden/fatigue and to account for scanner drift. A total of 202 brain images were acquired on the 3T scanner (see details below) in each run.

Face stimuli were selected from the FACES database (Ebner et al., 2010).^2^ We chose 32 young (age range: 18–31 years) and 32 older (age range: 69–80 years) neutrally expressive faces with equal numbers of male and female faces per age group. Faces were evenly split into two sets based on their age and gender, with eight faces per age-by-gender group in each run. Among each set of 32 face images, 16 faces (four from each age-by-gender group) were selected to create the scrambled face images (Figure 1; also see text footnote 2). In each run, the presentation order of the 48 face images (32 face and 16 scrambled face images) was pseudorandomized so that no more than three faces of the same age-by-gender group or three scrambled face images repeated in a row.

### Image Acquisition and Preprocessing

Brain images were acquired on a 3T Philips Achieva MR Scanner (Philips Medical Systems, Best, The Netherlands) with a 32-channel head coil. Whole-brain high-resolution three-dimensional T1-weighted anatomical reference images were acquired using an MP-RAGE sequence (sagittal plane, FOV = 240 × 240 × 170 mm; 1 mm^3^ isotropic voxels). Functional images were acquired using whole-head gradient-echo-planar imaging and single-shot gradient echo (38 interleaved slices, TR = 2 s, TE = 30 ms, FOV = 252 × 252 × 133 mm, flip angle = 90°, in-plane resolution = 80 × 80, no skip).

We applied standard preprocessing procedures using Statistical Parametric Mapping 12 (SPM 12; Welcome Department of Imaging Neuroscience). This included segmentation and spatial normalization into MNI space for structural images and slice time correction, realignment and unwarp, coregistration with structural data, spatial normalization into MNI space, resampled voxel size of 2 mm^3^, and smoothing with an 8 mm Gaussian kernel for functional images.

### Data Analysis

#### Categorization of Face Stimuli

Previous work had demonstrated that amygdala response was better predicted by consensus ratings (e.g., averaged ratings across a group of individuals) than by individual ratings (Engell et al., 2007). We therefore based the levels of face trustworthiness in our analyses on norm ratings (from an independent study by Pehlivanoglu et al., 2022)^3^ rather than on our study participants’ individual ratings.^4^ In particular, Pehlivanoglu et al. (2022) comprised 87 young (*M* = 30.98 years, *SD* = 4.46, 25–39 years, 28% females), 59 middle-aged (*M* = 51.91 years, *SD* = 6.09, 44–59 years, 64% females), and 47 older (*M* = 65.64 years, *SD* = 4.22, 60–78 years, 70% females) adults who rated the trustworthiness of each face in the FACES database (“*How trustworthy is this face?”)* on a scale ranging from 0 (“*Not at all trustworthy*”) to 100 (*“Extremely trustworthy”)*. Based on that data, we categorized the 64 face images used in the present study into five face trustworthiness levels.^5^

#### Behavioral Data

To test for age-related differences in face trustworthiness ratings (*Hypothesis 1*) and explore a potential oxytocin moderation on this effect, we conducted a repeated analysis of variance (ANOVA) on face trustworthiness ratings, with face trustworthiness level (categorical: 1 = least trustworthy to 5 = most trustworthy) as within-subject variable and age group (dichotomous: young vs. older) as well as treatment group (dichotomous: oxytocin vs. placebo) as between-subject variables.

#### Functional Magnetic Resonance Imaging Data

The functional brain data analysis comprised two steps. In the first step, we conducted a parametric analysis (Büchel et al., 1998) in SPM12 to determine voxels in the amygdala for which blood-oxygen-level-dependent (BOLD) signal was associated with face trustworthiness ratings. In particular, we created a first-level model for each participant. This model included zero-order regressors for the time series of face trials for each run. The model also included two first-order regressors (i.e., one regressor for each run) to capture the *linear trend* of face trustworthiness. We furthermore added 2 s-order regressors, orthogonal to the first-order regressors, to capture the *quadratic (U-shaped) trend* of face trustworthiness. In addition, the first-level model contained 16 regressors of no-interest: representing the time series of scramble face trials and the fixation for each run as well as six head movement parameters for each run. All regressors were convolved with the canonical hemodynamic response function (HRF). We used the general linear model approach (GLM; Friston et al., 1994) to calculate participant-specific parameter estimates pertaining to each regressor. The regressor estimates of the linear and the quadratic effects of face trustworthiness were used to create two corresponding second-level random effect models, respectively. We created two *t*-contrasts to identify brain activity associated with a significant negative linear and a significant positive quadratic effect, respectively.

Based on the literature, our hypotheses specifically focused on amygdala response associated with face trustworthiness. Therefore, we used small-volume correction of the *p*-values for the clusters in left and right amygdala that showed linear and/or quadratic effects of face trustworthiness. The amygdala mask we used for this small-volume correction was defined by the Automated Anatomical Labeling atlas (Tzourio-Mazoyer et al., 2002). For broader exploration of our dataset, we also conducted whole-brain analyses with a voxel-wise threshold of *p* < 0.05, FWE-corrected.

In the second step, we aimed to determine age-related differences in the associations between face trustworthiness level and BOLD activity (*Hypothesis 2* and *3*); as well as oxytocin moderation of these age-related differences. Thus, we created a first-level model, in which we defined regressors to represent the time series at each level of face trustworthiness per run (i.e., ten regressors in total for five levels of face trustworthiness and two runs). In addition, the model also consisted of 16 regressors of no-interest, including two for scramble faces, two for fixation, and twelve for head movement parameters. We then computed five *t*-contrasts to estimate BOLD signal change associated with each level of face trustworthiness. Using the Marsbar toolbox (Brett et al., 2002), we extracted parameter estimation of these *t*-contrasts, which represented the relative brain activation associated with each level of face trustworthiness, for each participant from clusters within the left and right amygdala that showed significant linear and/or quadratic effects of face trustworthiness. We conducted two multilevel regression models on these extracted parameter estimates, one for left and one for right amygdala, to determine age-related differences in the associations between face trustworthiness level and BOLD activity (*Hypothesis 2* and *3*); as well as oxytocin moderation of these age-related differences.

In each model, the face trustworthiness level and its quadratic transformation (which was orthogonal to the face trustworthiness level) served as within-subject predictors to model the linear and quadratic effects of face trustworthiness, respectively. Age group and treatment group, as well as their interaction, served as between-subject predictors in these models. We also considered the interaction between the linear and quadratic trends of face trustworthiness with age group and treatment group in the models, to assess the extent to which age group and/or treatment interacted with the linear and/or quadratic effects of face trustworthiness on amygdala activity. With reference to literature supporting sex-dimorphic effects of oxytocin (Lischke et al., 2012; Luo et al., 2017; Lieberz et al., 2020), including in aging (Ebner et al., 2015, 2016), in all analyses, participants sex (0 = male, 1 = female) served as a covariate.^6^

## Results

### Behavioral Results

**FIGURE 2:**
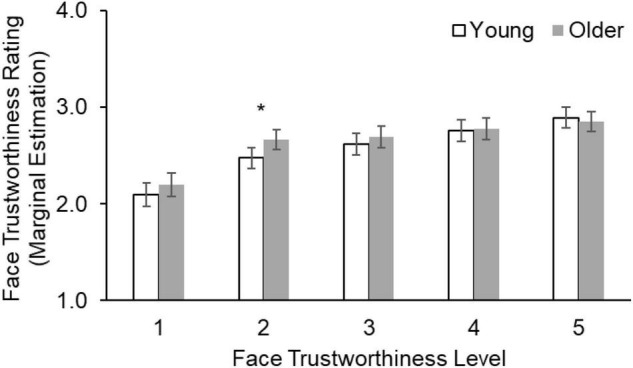
Face trustworthiness ratings (with higher scores indicating higher trustworthiness ratings) as a function of face trustworthiness level in young and older participants. Older compared to young participants gave higher trustworthiness ratings for “somewhat untrustworthy-looking” faces (face trustworthiness level 2). Error bars indicate 95% confidence intervals. *p < 0.05.

The Mauchly’s test for face trustworthiness level was significant, indicating that the assumption of sphericity was violated. Therefore, we reported any effects involving this variable with Greenhouse-Geisser corrected degrees of freedom. The main effect of age group was not significant [*F*_(1,__93)_ = 1.03, *p* = 0.31, η*_*p*_*^2^ = 0.01], suggesting no age-related differences in overall face trustworthiness ratings. The main effect of face trustworthiness level was significant [*F*_(3.01_, _280.25)_ = 70.98, *p* < 0.001, η*_*p*_*^2^ = 0.43], in that both young and older participants gave higher trustworthiness ratings to faces with higher levels of trustworthiness, indicating high consistency in trustworthiness ratings between the current sample and the independent norm sample (Pehlivanoglu et al., 2022). This main effect of face trustworthiness level was further qualified by a significant age moderation [*F*_(3.01, 280.25)_ = 3.68, *p* = 0.013, η*_*p*_*^2^ = 0.038]. In particular, older (*M* = 2.66, *SE* = 0.05) compared to young (*M* = 2.47, *SE* = 0.05) participants gave higher trustworthiness ratings to faces from the second level of face trustworthiness [*F*_(1, 93)_ = 6.46, *p* = 0.01, η*_*p*_*^2^ = 0.065], while the age groups did not differ in their ratings of the other four levels of trustworthiness (all *p*s > 0.10, Figure 2). This result partially supported *Hypothesis 1* in that older participants rated somewhat untrustworthy-looking faces as relatively more trustworthy than did young participants. No other main or interaction effects were significant for face trustworthiness ratings (all *p*s > 0.05; see Supplementary Material for parameter estimates of all variables).

### Neuroimaging Results

#### Regions of Interest Analyses

As shown in Table 2, one cluster in left amygdala showed a significant *negative linear effect* of face trustworthiness level, in that lower face trustworthiness ratings were associated with higher left amygdala activity. In addition, two clusters in the right amygdala showed significant *positive quadratic effects* of face trustworthiness level: that is, lower and higher compared to moderate face trustworthiness ratings were associated with greater right amygdala activity.

**TABLE 2:** Brain regions showing associations with face trustworthiness ratings across young and older participants.

	Peak voxel MNI coordinates			*P*-value		Cluster size
Region	*x*	*y*	*z*	Peak-level	Cluster-level	(# Voxels)
**Negative linear effect**						
*ROI analysis*						
Left amygdala	–24	–4	–24	0.004	0.01	19
*Whole-brain analysis*						
Bilateral cuneus/lingual gyri	14	–92	0	<0.001	<0.001	3,268
**Positive quadratic (U-shaped) effect**						
*ROI analysis*						
Right amygdala	32	–2	–18	0.003	0.008	32
	18	0	–18	0.017	0.018	7
*Whole-brain analysis*						
Medial pre-frontal cortex	–4	50	42	0.004	<0.001	133
Medial pre-frontal cortex	0	42	52	0.021	0.014	8
Left inferior/orbital frontal gyrus	–46	24	–12	0.002	0.002	33
Left superior temporal gyrus	–42	18	–30	0.003	0.009	12
Right middle frontal gyrus	38	14	40	0.002	0.001	50
Left middle frontal gyrus	–50	14	40	0.012	0.007	16
Left middle temporal gyrus	–48	2	–30	0.006	0.017	6
Right fusiform gyrus	26	–44	–16	0.011	0.007	15
Right supramarginal gyrus	60	–44	30	0.012	0.011	10
Right supramarginal gyrus/angular gyrus	50	–54	30	0.015	0.002	33
Left supramarginal gyrus	–58	–54	22	0.007	0.007	16
Left lingual gyrus	–12	–78	–12	0.003	<0.001	100
Left cuneus	–20	–96	–8	0.008	0.006	17

*The negative linear effect indicated that higher brain activity was associated with lower face trustworthiness ratings. The positive quadratic (U-shaped) effect indicated that higher brain activity was associated with both higher and lower (compared to moderate) face trustworthiness ratings. The brain regions, other than amygdala as ROI, are listed in this table from anterior to posterior. The amygdala findings are from the region of interest (ROI) analysis; all other findings are from the whole-brain analysis. We applied small volume correction to the p-values for clusters in amygdala and whole-brain correction for all other clusters. All p-values were FWE corrected. MNI, Montreal Neurological Institute.*

##### Left Amygdala

**FIGURE 3:**
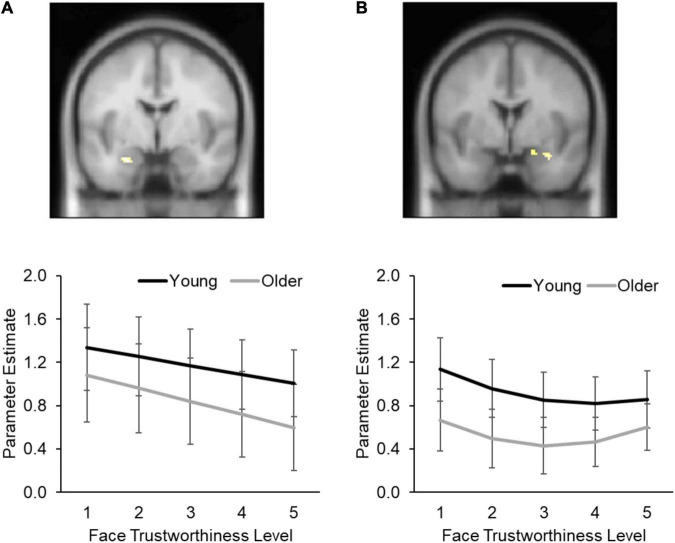
Amygdala response as a function of face trustworthiness in young and older participants for left **(A)** and right **(B)** amygdala. **(A)** In both age groups, left amygdala activity decreased with increasing face trustworthiness level (i.e., lower face trustworthiness ratings were associated with higher left amygdala activity; *negative linear effect*). **(B)** Also, for both age groups lower and higher compared to moderate face trustworthiness ratings were associated with greater right amygdala activity (*positive quadratic (U-shaped) association*); that is, lower and higher compared to moderate face trustworthiness were associated with greater right amygdala activity. In addition, only young participants also showed a negative linear effect of face trustworthiness level in right amygdala activity. Error bars indicate 95% confidence intervals.

Multilevel modeling analysis on the extracted beta values confirmed the significant negative linear effect of face trustworthiness level on left amygdala activity (*B* = –0.13, *z* = –3.55, *p* < 0.001, Cohen’s *f*^2^ = 0.04; Figure 3A). This negative linear effect in the left amygdala was not moderated by age group (*B* = 0.03, *z* = 0.63, *p* = 0.53, Cohen’s *f*^2^ = 0.001), thus not supporting *Hypothesis 2* in the left amygdala. No other main or interaction effects were significant for the left amygdala (all *p*s > 0.05; see Supplementary Material for parameter estimates of all variables).

##### Right Amygdala

Multilevel modeling analysis on the extracted beta values confirmed the significant positive quadratic effect of face trustworthiness level on right amygdala activity (*B* = 0.50, *z* = 1.98, *p* = 0.047, Cohen’s *f*^2^ = 0.02; Figure 3B). This positive quadratic effect in the right amygdala was not moderated by age group (*B* = 0.04, *z* = 1.09, *p* = 0.28, Cohen’s *f*^2^ < 0.001). This finding supported *Hypothesis 3* in the right amygdala, in that both young and older participants showed greater right amygdala activity for higher and lower face trustworthiness compared to moderate face trustworthiness (i.e., positive quadratic effect; Figure 3B).

There also was a significant negative linear effect of face trustworthiness level on right amygdala activity (*B* = –0.10, *z* = –3.26, *p* = 0.001, Cohen’s *f*^2^ = 0.01). This negative linear effect was further qualified by an age-group moderation (*B* = 0.09, *z* = 2.17, *p* = 0.03, Cohen’s *f*^2^ = 0.01), confirming *Hypothesis 2* for right amygdala. That is, young but not older participants showed greater right amygdala activity to faces for lower face trustworthiness (i.e., Figure 3B).

The interaction between age group, treatment group, and the linear effect of face trustworthiness was not significant (*B* = –0.08, *z* = –1.09, *p* = 0.28, Cohen’s *f*^2^ = 0.003), thus not supporting an oxytocin moderation on age-related differences in the negative linear association between face trustworthiness and right amygdala activity. No other main or interaction effects were significant for right amygdala (all *p*s > 0.05; see Supplementary Material for parameter estimates of all variables).

#### Whole-Brain Exploratory Analysis

An exploratory whole-brain analysis showed a negative linear effect of face trustworthiness level on activity in a cluster encompassing bilateral lingual gyrus and cuneus in both age groups. In addition, a positive quadratic effect of face trustworthiness level on brain activity was observed in various regions including the medial prefrontal cortex, bilateral middle frontal gyri, left superior temporal sulcus (e.g., superior and middle temporal gyrus), bilateral supramarginal gyrus, left lingual gyrus, left cuneus, and right fusiform gyrus (Table 2).

## Discussion

This study examined age-related differences in face trustworthiness evaluation and associated amygdala activity and probed the moderating role of a single dose of intranasal oxytocin on these effects. The study generated three main findings. First, older compared to young participants rated somewhat untrustworthy-looking faces as more trustworthy (while the age groups gave comparable face trustworthiness ratings overall). Second, young participants showed greater bilateral amygdala activity to untrustworthy- relative to trustworthy-looking faces, but this effect was only present in left but not in right amygdala in older participants. Furthermore, both age groups showed a positive quadratic (U-shaped) association between right amygdala activity and face trustworthiness ratings, in that both high and low (relative to moderately) trustworthy-looking faces were associated with greater right amygdala activity. Third, in this study, intranasal oxytocin did not moderate face trustworthiness ratings or associated amygdala activity in either of the age groups. Next, we discuss empirical and theoretical implications of these novel findings.

### Higher Trustworthiness Ratings for Ambiguously Untrustworthy-Looking Faces in Older Than Young Adults

Our finding that older compared to young adults evaluated somewhat untrustworthy-looking faces as more trustworthy is in line with emerging evidence of decreased sensitivity to deceptive cues in aging (Ruffman et al., 2012; Frazier et al., 2021; see Ebner et al., 2022, for an overview). This finding also suggests that the age-related positivity effect (Reed et al., 2014) applies to face trustworthiness evaluation (see also Zebrowitz et al., 2013, 2017), in that older adults rated untrustworthy-looking faces, as faces depicting negative facial cues, as less negative/more positive than young adults did.

Previous studies have reported comparable findings (i.e., relatively more favorable ratings for untrustworthy-looking faces from older than young adults; Castle et al., 2012; Zebrowitz et al., 2017; Cassidy et al., 2019; see also Bailey et al., 2016). Our study importantly qualifies this previous work by demonstrating that higher trustworthiness ratings were only given for somewhat untrustworthy-looking faces, but not for very untrustworthy-looking faces. While both signal negative facial cues, somewhat untrustworthy-looking faces display ambiguous (negative) facial cues. Thus, it is possible that the positivity effect in aging for face trustworthiness is selective; that is, older adults may be as sensitive as young adults to negative/deceptive cues in non-ambivalent faces, but not in ambivalent faces.

In fact, supporting this interpretation, ambivalent information requires rather complex cognitive operations such as interference resolution (e.g., attending to specific aspects of a stimulus while ignoring (salient) other aspects; Stanley and Blanchard-Fields, 2008; O’Connor et al., 2019). Given age-related decline in working memory (Pehlivanoglu et al., 2014; for a meta-analysis see, Bopp and Verhaeghen, 2007) combined with age-related reduction in sensitivity to deceptive cues (Castle et al., 2012) and an increased positivity effect with age (Carstensen and Mikels, 2005), older participants may have had difficulty in focusing on cues of untrustworthiness while filtering out cues of trustworthiness in ambiguously untrustworthy-looking faces. In line with this explanation, other studies showed that older compared to young adults used fewer negative words to describe ambiguous scenarios (Juang and Knight, 2016; Mikels and Shuster, 2016) and gave more positive evaluations to ambiguous facial expressions (i.e., surprise; Neta and Tong, 2016; Neta et al., 2018).

This age-related reduced differentiation when confronted with ambiguously negative (untrustworthy) cues may be reflective of older adults’ perceptual dedifferentiation (Park et al., 2012) and/or may reflect a shift in decision criteria in trust-related decision making. That is, when cues of untrustworthiness are not distinct, older adults may not pick up on them, and perhaps particularly so for negative cues. In support of this interpretation, age-related dedifferentiation in face emotion recognition is greater/or only occurs for negative (e.g., anger, disgust) but not positive or neutral expressions (Franklin and Zebrowitz, 2017).

### Lateralization and Age Effects in the Association Between Amygdala Response and Face Trustworthiness

Consistent with previous findings in young adults (Said et al., 2009; Freeman et al., 2014; see Santos et al., 2016, for a meta-analysis), we identified a complex pattern of amygdala response to face trustworthiness in our age-heterogeneous sample, which qualifies prior work (Said et al., 2009). In particular, we observed a lateralization in amygdala response to facial trustworthiness that was further moderated by age, as discussed next.

Comparable across both age groups, right amygdala response was greater to more and less trustworthy-looking than to moderately trustworthy-looking faces (i.e., U-shaped association). In left amygdala, in contrast, we observed a negative linear association; in that left amygdala activity decreased with increasing face trustworthiness. Right amygdala has been shown to mediate unconscious processing of emotional information, while left amygdala has been associated with conscious processing of emotional stimuli (Morris et al., 1998, 1999; Allen et al., 2021). Also, a U-shaped amygdala response to face trustworthiness has been observed in implicit tasks (Mattavelli et al., 2012; Rule et al., 2013) or when stimuli were presented so briefly (e.g., 200 ms) that elaborate processing was not possible (Freeman et al., 2014). Mapping onto these previous findings, the U-shaped association between right amygdala response and face trustworthiness ratings in our study may reflect more implicit processing, while the negative linear association between left amygdala response and face trustworthiness ratings may reflect more explicit processing.

Further, supporting an age-differential response pattern in right amygdala, we found that young but not older participants showed a negative linear trend in addition to the U-shaped association. As a result, young but not older participants showed enhanced right amygdala activity to less untrustworthy-looking faces. As amygdala response toward face trustworthiness may reflect the level of social salience (Sander et al., 2003; Vuilleumier, 2009), which further signal approach/avoidance behavior (Todorov, 2008; Todorov and Engell, 2008), age-differential linear response in right amygdala could be interpreted as suggesting greater salience of untrustworthy-looking faces among young but not older adults (or at least, that salience of untrustworthy-looking faces is reduced among older relative to young adults). Thus, our amygdala findings are in accord with brain data on the positivity effect suggesting reduced response to negative but not positive information (Mather et al., 2004; see also Reed and Carstensen, 2012, for an overview).

Visual inspection of the two clusters identified in left and right amygdala suggested their location in the lateral nuclei, known as the gateway of amygdala, that receive perceptual input (LeDoux, 2007). Stronger magnetic field imaging such as with a 7T scanner in future studies will allow enhanced localization (Vu et al., 2017) and facilitate interpretation of the processes underlying face trustworthiness evaluation in young and older adults.

### Oxytocin Did Not Modulate Face Trustworthiness Evaluation in Brain or Behavior

In this study we found no evidence of a moderation of face trustworthiness evaluation by intranasal oxytocin, neither in behavior nor in brain response. Oxytocin has been promoted as crucial modulator of social cognition and behavior (Harari-Dahan and Bernstein, 2014; Quintana et al., 2018), including in aging (Horta et al., 2020), and has been discussed in the context of trustworthiness perception (Van IJzendoorn and Bakermans-Kranenburg, 2012). However, some empirical work does not support such effects (Lambert et al., 2014; Grainger et al., 2018; see also Declerck et al., 2020). The present study findings align with studies that do not support an effect of a single dose of intranasal oxytocin on face trustworthiness evaluation and extend this null effect to older adults (see Grainger et al., 2019).

There are various possible explanations for the observed non-significant effects of oxytocin on face trustworthiness in brain and behavior. The present study used faces with neutral expressions, which may have reduced emotional reaction/approach-avoidance tendencies to the images. Some of the previous studies that found social-cognitive effects of oxytocin used strong social (e.g., in-group vs. out-group; De Dreu et al., 2010) and emotional (e.g., emotional expression; Horta et al., 2019) manipulations in their experimental designs. It is possible that stimuli with affluent social and emotional content facilitate and/or enhance oxytocin’s social-cognitive effects.

Also, the majority of faces used in the present study fell somewhere in the middle of the trustworthiness spectrum, as they were retrieved from a naturalistic set of images taken from real people (see details about the FACES database in Ebner et al., 2010). The present study’s 4-point rating scale with two extreme, opposite ends (i.e., *very untrustworthy* to *very trustworthy*) may have had limited sensitivity to differentiate along trustworthiness among this set of naturalistic images and thus also limited our ability to model the BOLD signal along this dimension. For example, with a comparable sample size of 96 participants who underwent a single dose of either intranasal oxytocin or placebo and using neutral faces as stimuli but a 7-point trustworthiness rating scale, Theodoridou et al. (2009) found higher face trustworthiness ratings in the oxytocin than the placebo group. Future studies will be able to test if such methodological differences underlie variability in detecting oxytocin effects on face trustworthiness evaluation.

Moving forward, studies with a larger sample size are warranted to follow up on the oxytocin null effects observed in the present study. In fact, a recent meta-analysis (Leppanen et al., 2017), which was published after completion of the present data collection and suggested that for a study with oxytocin vs. placebo administration as between-subject factor a sample size of 64 participants per treatment group is necessary to obtain sufficient power (80%) to detect a moderate effect of oxytocin on social cognition (see Supplementary Material for details regarding the interaction we observed between treatment group and face trustworthiness level, which were not statistical significant as per our alpha level cut off, but approached significance). While randomized for treatment group assignment, the present study used a between-subject design and all measures were collected after treatment administration. Future extension of this work would benefit from a cross-over design to account for possible individual differences at baseline. In fact, following recent recommendations (Naselaris et al., 2021), within-subject designs are better suited as their higher sampling of a more limited number of subjects compared to the smaller sampling of a larger number of individuals in a between-subject design are more powerful to detect significant effects. This emphasis on depth of assessment in individual brains over breadth of assessment among multiple brains is also beneficial for enhancing resolution and the signal-to-noise ratio in neuroimaging analysis, and thus a fruitful path forward.

## Conclusion

The present study extends previous work by demonstrating age-related differences in face trustworthiness ratings for ambiguously untrustworthy-looking faces. The study also demonstrates lateralization as well as age effects in amygdala response underlying face trustworthiness evaluation. In sum, this work supports the notion of reduced sensitivity/dampened response to cues of untrustworthiness in aging and supports an extension of the age-related positivity effect to the domain of face trustworthiness evaluation. Finally, the paper adds to a growing literature of possible null effects of single-dose intranasal oxytocin administration on face trustworthiness perception.

## Data Availability Statement

The raw data supporting the conclusions of this article will be made available by the authors, without undue reservation.

## Ethics Statement

The studies involving human participants were reviewed and approved by the University of Florida Institutional Review Board. The patients/participants provided their written informed consent to participate in this study.

## Author Contributions

TL: conceptualization, methodology, data collection, formal analysis, writing original draft, and revising. DP: conceptualization, data collection, writing original draft, and revising. MZ: methodology and revising. PL: methodology, writing original draft, and revising. AW, DF, and HF: conceptualization, methodology, and revising. NE: conceptualization, methodology, formal analysis, writing original draft, and revising. All authors contributed to the article and approved the submitted version.

## Conflict of Interest

The authors declare that the research was conducted in the absence of any commercial or financial relationships that could be construed as a potential conflict of interest.

## Publisher’s Note

All claims expressed in this article are solely those of the authors and do not necessarily represent those of their affiliated organizations, or those of the publisher, the editors and the reviewers. Any product that may be evaluated in this article, or claim that may be made by its manufacturer, is not guaranteed or endorsed by the publisher.

## Footnotes

1 A total of 7 participants (4 young; 3 older) did not complete the functional magnetic resonance imaging (fMRI) task due to technical issues, time limitations, or drop out after the screening session.

2
https://faces.mpdl.mpg.de/imeji/

3
https://psyarxiv.com/rknu2/

4 The correlation between the present study’s subjective face trustworthiness ratings and the face trustworthiness ratings (norm ratings) from Pehlivanoglu et al. (2022) was high (*r* = 0.72), supporting strong convergence across the two studies.

5 This approach allowed us to model both linear and quadratic effects of face trustworthiness on brain activity, while maintaining sufficient number of trials within each face trustworthiness level for robust estimation.

6 We conducted parallel analyses with sex as a moderator. None of the interactions with sex were significant, thus not supporting a moderation effect of sex on the results reported here.
