# Supplementary material for: Age-Related Differences in Amygdala Activation Associated With Face Trustworthiness but No Evidence of Oxytocin Modulation
Source: Front Psychol. 2022 Jun 23;13:838642. doi: 10.3389/fpsyg.2022.838642 (PMC9262048; doi:10.3389/fpsyg.2022.838642)
Supplement: Supplementary file 1 [file Data_Sheet_1.docx]

**Supplementary Materials to Age-Related Differences in Amygdala Activation Associated with Face Trustworthiness but No Evidence of Oxytocin Modulation by Lin, Pehlivanoglu, Ziaei, Liu, Woods, Feifel, Fischer, & Ebner**

***Behavioral Results: Effects of Treatment Group (Oxytocin vs. Placebo) on Face Trustworthiness Ratings***. The interaction between treatment group and face trustworthiness level on face trustworthiness ratings was not significant [*F*(3.01, 280.25) = 2.22, *p* = 0.086, *η*_p_^2^ = 0.023].


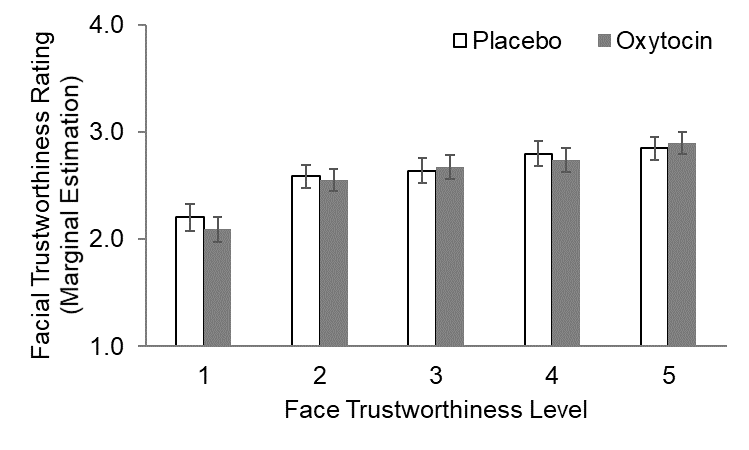


*Figure S1.* Face trustworthiness ratings (with higher scores indicating higher trustworthiness ratings) as a function of face trustworthiness level in the placebo and oxytocin groups. Error bars indicate 95% confidence intervals.

Table S1. *Marginal Estimates [Mean (Standard Error)] of Face Trustworthiness Ratings by Face Trustworthiness Level for Young and Older Participants in the Placebo and Oxytocin Groups.*

|  | **Young** | | **Older** | |
| --- | --- | --- | --- | --- |
| **Face Trustworthiness Level** | **Placebo** | **Oxytocin** | **Placebo** | **Oxytocin** |
|  | Mean (*SE*) | Mean (*SE*) | Mean (*SE*) | Mean (*SE*) |
| 1 | 2.10 (*0.09*) | 2.09 (*0.08*) | 2.31 (*0.09*) | 2.09 (*0.08*) |
| 2 | 2.46 (*0.08*) | 2.49 (*0.07*) | 2.72 (*0.08*) | 2.61 (*0.07*) |
| 3 | 2.62 (*0.08*) | 2.61 (*0.08*) | 2.65 (*0.08*) | 2.73 (*0.08*) |
| 4 | 2.77 (*0.08*) | 2.75 (*0.08*) | 2.82 (*0.08*) | 2.73 (*0.08*) |
| 5 | 2.87 (*0.08*) | 2.91 (*0.07*) | 2.83 (*0.08*) | 2.88 (*0.07*) |

Note. SE = Standard Error.

***Neuroimaging Results: Effects of Treatment Group (Oxytocin vs. Placebo) on Left Amygdala Activity Associated with Face Trustworthiness***. The interaction between treatment group and the linear effect of face trustworthiness on left amygdala activity was not significant (*B* = 0.10, *z* = 1.65, *p* = 0.098). Please see Table S2 for all effects from the multilevel modeling analyses on left and right amygdala activity.

Table S2. *Results of the Multilevel Linear Regression for Age Group, Treatment Group, and the Linear/Quadratic Trends of Face Trustworthiness on Left and Right Amygdala Activity*

|  | **Left Amygdala** | | | **Right Amygdala** | | |
| --- | --- | --- | --- | --- | --- | --- |
| **Fixed Effect** | B (SE) | [95% CI] | B (SE) | | [95% CI] |  |
| Age Group | -0.25 (0.42) | [-1.08, 0.57] | -0.71 (0.32) | | [-1.33, -0.09] |  |
| Treatment Group | 0.28 (0.44) | [-0.59, 1.15] | -0.0003 (0.32) | | [-0.63, 0.63] |  |
| Age Group × Treatment Group | 0.06 (0.65) | [-1.21, 1.32] | 0.27 (0.46) | | [-0.63, 1.17] |  |
| Face Trustworthiness (Linear) | **-0.13 (0.04)** | **[-0.21, -0.06]** | **-0.1 (0.03)** | | **[-0.16, -0.04]** |  |
| Age Group × Face Trustworthiness (Linear) | 0.03 (0.06) | [-0.07, 0.14] | **0.09 (0.04)** | | **[0.01, 0.18]** |  |
| Treatment Group × Face Trustworthiness (Linear) | 0.1 (0.06) | [-0.02, 0.21] | 0.06 (0.05) | | [-0.03, 0.16] |  |
| Age Group × Treatment Group × Face Trustworthiness (Linear) | -0.14 (0.09) | [-0.31, 0.04] | -0.08 (0.07) | | [-0.21, 0.06] |  |
| Face Trustworthiness (Quadratic) | 0.03 (0.04) | [-0.06, 0.11] | **0.05 (0.03)** | | **[0.001, 0.10]** |  |
| Age Group × Face Trustworthiness (Quadratic) | 0.02 (0.06) | [-0.1, 0.14] | 0.04 (0.03) | | [-0.03, 0.11] |  |
| Treatment Group × Face Trustworthiness (Quadratic) | -0.04 (0.05) | [-0.15, 0.07] | -0.03 (0.03) | | [-0.09, 0.04] |  |
| Age Group × Treatment Group × Face Trustworthiness (Quadratic) | -0.02 (0.09) | [-0.2, 0.16] | -0.04 (0.05) | | [-0.14, 0.06] |  |
| Sex | 0.05 (0.27) | [-0.48, 0.57] | 0.11 (0.18) | | [-0.24, 0.45] |  |
| Intercept | **1.25 (0.32)** | **[0.61, 1.89]** | **1.08 (0.24)** | | **[0.61, 1.55]** |  |
|  |  |  |  | |  |  |
| **Random Effect** | σ^2^ (SE) | [95% CI] | σ^2^ (SE) | | [95% CI] |  |
| Intercept | **1.58 (0.28)** | **[1.11, 2.23]** | **0.68 (0.10)** | | **[0.51, 0.90]** |  |

Note. **Bold print** indicates significant effects at *p* < 0.05.
